# Mourning and Management of the COVID-19 Health Emergency in the Priestly Community: Qualitative Research in a Region of Northern Italy Severely Affected by the Pandemic

**DOI:** 10.3389/fpubh.2021.622592

**Published:** 2021-02-12

**Authors:** Ines Testoni, Silvia Zanellato, Erika Iacona, Cristina Marogna, Paolo Cottone, Kirk Bingaman

**Affiliations:** ^1^Department of Philosophy, Sociology, Education, and Applied Psychology, University of Padova, Padua, Italy; ^2^Emili Sagol Creative Arts Therapies Research Center, University of Haifa, Haifa, Israel; ^3^Graduate School of Religion and Religious Education, Fordham University, New York, NY, United States

**Keywords:** COVID-19 pandemic, priest, traumatic loss, mourning, funeral, religiosity

## Abstract

**Background:** The COVID-19 pandemic is causing major social changes to which significant psychological effects are linked. During the first phase of the pandemic wave in Italy, whilst there was insufficient information about the phenomenon and the strategies to safeguard the population against it, many categories of people, whose professions required constant contact with the public, were affected by the contagion.

**Aims:** The literature has shown how religiousness can support the management of stress due to diseases and health risks. In relation to this, the current study wanted to investigate how priests managed the early stages of the pandemic. This work, therefore, aimed to investigate the psychological experiences related to the contagion and the eventual death of colleagues as well as the resilience strategies activated by the priests during the process.

**Participants:** The research involved 12 Catholic priests, all male and aged between 42 and 63 years. They came from the same pastoral community in one of the regions in Northern Italy that were most affected during the first phase of the pandemic. Those ministers had been constantly in contact with the faithful of their parishes since the breakout of the virus.

**Methodology:** A qualitative research design was adopted, and in-depth interviews were conducted. The dialogues aimed at investigating the deep, personal and relational experiences of the priests, together with their concerns and the tools they adopted to manage anxiety. The texts obtained from the interviews were subjected to thematic analysis.

**Results:** The areas studied concerned the experiences of the participants during the lockdown, the implications of social distancing and lack of funeral rituality and, finally, the importance of prayer as a resilience factor.

**Conclusions:** In the current scenario dominated by the pandemic, it is significant and stimulating to understand and reflect on the functions and roles of the experiences of faith, particularly the act of elaborating the process of mourning due to COVID-19.

## Introduction

Literature has already considered the importance of the spiritual support work of priests in their communities and how their activity exposes them to difficulties and critical incidents, especially when they have to operate in difficult contexts (1–4). The COVID-19 pandemic has strongly contributed to the deterioration of the general level of mental health and well-being in all countries where the pandemic exploded (5). In fact, it has intensified the feelings of loneliness and social isolation due to the lockdown and the need for distancing as well as the rarefaction of social relations to prevent the spread of the contagion (6, 7). In Italy, the first phases of the COVID-19 pandemic strongly affected many categories of people who, either for professional or personal reasons, had to be in daily and direct contact with a significant number of other individuals (8, 9). In addition to medical and nursing staff and those working in commercial distribution, these groups of people also included priests (10). In normal times, when death appears in family life, mourners reflect on their lives and on their close relationships with relatives and with God. In these tasks, they are often assisted by priests, so that they can open their mind and face suffering, forgiveness and the perspective of the afterlife. In the emergency phase, during the breakout of the pandemic and in the subsequent lockdown, the manner in which the numerous deaths occurred exposed the survivors to traumatic loss, and priests were called upon to help them in various and exceptional ways. Unfortunately, these events exposed the priests to the risk of contracting the virus even without being guaranteed a really effective form of support due to the dramatic and unmanageable situation. Indeed, during the COVID-19 breakout, people who suffered from the death of their loved ones had to face the factors that classically characterize traumatic loss, such as sudden death and the manner in which the death was reported.

Moreover, social loneliness, brought about by government-imposed distancing to prevent transmission, heightened the emotional loneliness caused by the breaking of an emotional bond, thereby exacerbating its negative effects as the literature on mourning had already described (5, 11–13). For families, their COVID-19 experiences involved anxiety, separation, loss, grief and financial distress, and recent research showed how the pandemic exposed the world population to risk factors related to chronic psychological distress (14, 15). The literature emphasizes the need to carry out studies that consider the clinical risks for traumatic mourning caused by the pandemic (16). Aside from all these dramatic factors, the importance of other dimensions that exacerbated mourners' suffering, including the inability to accompany their loved ones during the end of life and the inability to celebrate the funeral rites, emerged (17, 18). These limitations restricted the possibilities of resorting to religion as a tool for the elaboration of suffering.

The importance of funeral services has been addressed by numerous studies highlighting their relevance both from the point of view of the mourners and the community [e.g., (19)]. The elaboration of loss through a religious rite offers a consolation that buffers the pain brought on by such a loss and makes it bearable (20). Scholars already showed how spirituality and religiosity can help develop resilience in stressful situations related to health and grief (21–23). These two aspects, whilst being able to coexist, are not limited to each other: in fact, spirituality is, in itself, independent of any external regulation of a religious nature and concerns the ability of the individual to access the dimension of transcendence (24). The tools offered by religion are based on the social sharing of rituality, language and symbolism (25), which directly influence a person's ability to manage experiences of death and mourning (26). The predictive power of religious commitment to well-being, both physical and psychological, could be explained by the affective relationship established between the believer and divinity. Such a bond could be based, like all feelings of union, on the need for protection and security to face the terrifying prospect of death (27–31). The darkening of spirituality and religiosity implies the nullification of the consolatory potential offered by these dimensions in the management of illness and loss (21, 22).

Yet, the literature seems to have failed to consider the point of view of priests, as no research has yet to investigate how the stress linked to the impossibility of officiating funerals due to emergency situations may have affected their well-being. To remedy such a gap in the literature, this article aims to explore the role of pastoral care during the pandemic and examine how priests managed their chaplaincy during this time.

## Aims of the Study

The present study aimed to analyse the emotional experiences of priests belonging to a community in Northern Italy, which had been strongly plagued by the first two phases of the COVID-19 pandemic. The main objective was to listen to the priests in order to understand how they had experienced the first phase of the pandemic, which coincided with the general lockdown (March 11 to May 3, 2020). It was important to explore whether and how they had been influenced by the awareness that many priests had been infected and that their category had been recognized as an at-risk group. We further wanted to study how they experienced the limitations on the possibilities of officiating funeral rites and how they managed this limitation with the mourners within the community of believers. In parallel, we sought to consider whether and how their representations of death affected their pastoral activities and contact with the faithful and how they perceived the role of religion in the management of collective trauma amongst believers.

## Participants and Methodology

This research followed a qualitative research design, particularly the assumptions of the phenomenological interpretative analysis or IPA (32). The study was conducted at the beginning of phase 2 of the pandemic (May 2020) and involved 12 participants, all members of pastoral communities in one of the regions in Northern Italy most affected by the pandemic during the first phase. The participants were aged between 42 and 63 years (average = 54 years; SD = 7) and were living with other priests and/or seminarians. Before starting, the purpose and the modalities of the research were explained to all the participants.

The data collection was carried out through in-depth interviews, which allowed the researchers to understand the participants' points of view on the investigated topic and draw on in-depth narratives about their experiences, attitudes and perceptions (20). The dialogues, lasting about 60 min each, were realized via the Internet; these were recorded and literally transcribed. The peculiar design of the IPA allowed the researchers to obtain a general and flexible outline, which left the possibility of modifications according to the progress of the dialogues with each participant. In this way, the themes they considered most significant can be further explored. This methodology was chosen, because it gave access to the sphere of profound experiences, thus responding to the need of examining how people made sense of their lived experiences (32–35). Therefore, the analysis of these experiences made it possible to understand how participants related to the COVID-19 pandemic.

As required by the approach, we followed three different levels of analysis of the texts: the idiographic analysis (repeated listening to the audio recordings of the individual interviews and reading of their transcription, in order to retrieve as much information as possible about how the participants lived and managed their experiences, thus leading to the recognition of the main nodes of their narratives), interpretation (recognition of the meanings of the main nodes and their links with the whole discourse, recognition of the perspectives of each individual participant as well as the similarities and the differences between them), and phenomenological dimension (recognition of a unitary integrated discourse that emphasizes individual specificities representing specific but also shared points of view). This procedure allowed the researchers to highlight the main points of the narrative through the faithful transcription of the interview and the rereading of the text (36). Next, we proceeded with the analysis of the semantic content and the used language as well as the identification of the themes that emerged. Finally, the various themes were organized in clusters and then in superordinate categories, allowing the researchers to achieve a more articulated analysis of the information collected.

It has to be noted that this type of analysis does not aim at grasping the essence of the phenomenon, but only at bringing out the most relevant themes reported by the participants (33). The analysis process was meant to facilitate the identification of some fundamental themes emerging from the narrations through a bottom-up approach. These themes, which were common to several participants, were grouped into broader thematic categories (37). Analyses were performed by a trained researcher under the supervision of an experienced qualitative analysis researcher. After they reached agreement on the interpretation, another researcher discussed the procedure and the first results obtained. Finally, after modifications agreed upon by the three researchers, the final structure of the report was defined.

Informed consent was obtained from all the participants. The research has been approved by the Ethics Committee of the University of Padua with unique number CD8470F7F42ACDD0679DC171884311B3 and protocol number 3635. As requested by ethical norms, all the names reported below are fictitious to respect the anonymity of the participants.

## Results

During the meetings, it was possible to divide the experiences reported into different thematic nodes related to the following: the emotions experienced during the initial emergency phase, the implications of social distancing and the lack of funeral rituality and the importance of prayer as a factor of resilience.

### First Theme: The Prevailing Early Emotions

The participants reported a concentration of negative emotionality associated with fear, uncertainty and helplessness, mainly during the first weeks of isolation. Domenico, a 58-year-old man, has been a priest for 33 years and spent 24 of them in a mission in Kenya. He had been the pastor of a community of about 4,000 people for 6 years, and the priest with whom he lived at that time had just died after a long illness. “Having to deal with the death of the colleague I was living with,” he said, “made me feel an abysmal emptiness. This was associated with the emptiness of the lockdown, because suddenly, I no longer had the possibility to see, to feel, to organize activities with people… how relevant can I be as a priest?” Franco, 53 years old, a priest for 27 years and a co-pastor for 16 years of a community of about 7,500 people with another priest, described something similar:

I don't think I have ever experienced real fear and fright, but I was overwhelmed by questions about the future. I was constantly asking myself what would happen in the future and what tools I had to face the most fearsome scenarios.

Michele, a 61-year-old man who had been the parish priest of a community of about 9,000 people for 11 years and who, at the time of the interview, was living with his mother shared:

I was assailed by an irrational fear, what happens when you are faced with something dangerous, an enemy. A killer who moves in an ambush and you can't predict if he will strike from the air, from the ground, if he will take your feet, your hands, because you don't know anything about him, you don't know anything. All these have been strongly destabilizing.

Michele further expressed his experiences:

Here, the emotions were fear and disenchantment and dismay. I wondered how I, my mother [and] my flock would react. I did not know what to propose to the community that I imagined to be as disoriented as I was. What I missed most were the social relationships, all the groups, the pastoral council, the meetings with the altar boys, all the celebrations I had planned: masses, confirmations, confessions, prayers for the spiritual journey. Suddenly the fear of evil, of the unknown evil that paralyzes me, fell upon me. For a moment, I was overwhelmed by the terror of losing my faith. The first time I saw the church empty I cried and I felt lost as the parishioners sent me messages asking me “What is going on?” “What is the point of all this?” I felt that they really needed me as a point of reference and that my bewilderment made them miss it, together with the community, when they met to celebrate the Eucharist. So, I asked myself how I could overcome my helplessness. I don't know how to use social networks, not everyone uses the phone or the computer. How could I reach them? Luckily, some parishioners helped me and on the Facebook page of the parish, we started broadcasting the Sunday Mass. The first time was a unique emotion. I saw people coming out of the balconies to say goodbye. Every Sunday during the mass, I called them by name, those of the Christian association, group by group, and they answered “Here I am!”

This first phase of personal fear was followed by a second one, in which attention to other people and worries for the neighbors became dominant. The desire to transform the time of social closure into a moment of deep reflection characterized the narratives of all the participants. As depicted by Patrizio, a 56-year-old parish priest who had served for 31 years in a community of about 4,500 inhabitants and had been living with a colleague:

At a certain point, I overcame my personal anxieties and remembered that, first of all, my role is to have a responsibility toward others. I thought that I had to help those who were not able to cope and to think also for those who did not know how to manage this situation.

Mauro, a 55-year-old priest for 30 years and pastor for 16 years of a community of 4,000 people, linked the experience of COVID-19 to the sacred texts:

It is important to remember that every crisis implies a change that can be an improvement. The exile for the people of Israel was an immense tragedy, which occurred because of the destruction of Jerusalem […] And yet, if we think about it, that great suffering was paradoxically very fruitful and allowed the Old Testament, which begins precisely the exile, to take shape entirely. What was announced as a period of great pain and misfortune turned out to be the great and fruitful teaching of the Old Testament.

### Second Theme: Social Distancing and The Perceived Inadequacy of The Funeral Rituality

According to the participants, the pandemic caused people to experience strong feelings of loneliness accompanied by deep sadness and the need to safeguard the quality of human relationships. The lack of social contact was a source of suffering for everyone, but “we must not make it become social distance or fracture [or even] estrangement from each other” as Domenico stressed. During this period, an ardently intense desire to return to the community also emerged, thus strengthening the need—according to Franco—to “build a bridge” and extend individual and private reflection to the entire community:

At this time, more than ever, I try to transform my dialogue with God into an effective social relationship. Now, more than ever, I feel that my faith call must express itself in social actions that help people to overcome this trauma.

Giacomo, a 61-year-old pastor of a community of about 7,000 faithful, continued in this thematic line, underlining how the symbolic Eucharistic action and the meeting during religious services are “the construction of the social body, which requires to feel united and aggregated to react to the trauma, also through prayer.” Giacomo, who had been living with an Ecuadorian colleague, also maintained this level of analysis as “unliveable.” This position is shared by Stefano, a 42-year-old man who has been an assistant parish priest of a parish of 7,500 inhabitants for 8 years. He said that the technological tools, although useful, have not completely made up for the lack of warmth and affection:

Man is relationship, he needs a direct dialogue. He needs to feel closeness and affectivity. The contact, for example, of the handshake makes the presence felt, it is not just a simple greeting said with the voice. Communication mediated via computer or other technological means reduces this type of presence. In this way, the soul of the relationship is missing.

Regarding the difficulties caused by the lockdown and social distancing, Domenico explained how the Internet has helped:

We are immediately equipped not to remain isolated in our task […] We always guaranteed the essential interventions, first in deferred ways and then by streaming with the means we had at that time. It was not easy, because the parishes were not very well equipped. However, we—the priests—went online and got informed about what everyone was doing on the Internet. I managed to create a WhatsApp group by inviting everyone to sign up in order to receive links and necessary information. Other colleagues have created listening groups. Now, the WhatsApp group has 238 members and we discuss issues in various communities and neighboring villages. We have proposed the *Via Crucis* (Way of the Cross) on Friday and organized the masses on Sunday. Choirs have been set up for Sunday songs and also a real musical company has been set up: there are those who sing, those who record, those who mix, those who take care of the audio effects. Those were all important experiences. For example, after she saw her daughter engaged in these operations, an elderly woman who usually watched Mass doing her house crafts felt more involved and is now participating in the streaming Mass whilst standing still, because she is now more involved.

Alessandro continued along the same line:

We have also activated online catechism and a distance education activity, from kindergarten to high school. Honestly, it was not easy for us to organize all these or to involve children and adolescents, because they need real closeness, to meet in a concrete space in which to move and act. Yes, everything is much more difficult, but let's try to make it.

However, it was the initial phase of the pandemic that outlined what would have been the most traumatic consequence of the social distancing: the impossibility of accompanying the dying for the last farewell, culminating in the prohibition of officiating the funeral rite in the traditional way. As Mauro said:

It was traumatic for the faithful not having the necessary space to deal with the death of their relatives. From the way I perceived their pain, they all felt as if something extremely precious had been stolen from them. What was taken away from them is precisely the path of processing the loss, and this made them unable to make sense of the void. They live with the feeling that absence is a presence.

Simone, a 63-year-old pastor for 38 years of a community of about 1,100 inhabitants, and who had been living with four other priests, continued this theme, saying that:

The emptiness and the sense of loss have been amplified by the impossibility of giving body to the community that gathers around the pain with prayer. The funeral rite guarantees this presence and helps give meaning to death and loss. We have all found ourselves in front of a void without being able to attribute meaning to it; therefore, we face a great question mark and are forced to ask ourselves what is beyond death in solitude, without the chorus of the community that gathers around this mystery.

Indeed, the loneliness associated with these rituality-related shortcomings had an effect that Franco called “a lacerating lack.” However, according to Giacomo, “The mourners, during the limited funeral resulting from the ongoing emergency, showed great civic sense and did not present recriminations. They seemed very aware.” Nicola, a 42-year-old man who had served as the pastor of a community of about 3,000 people for 12 years, linked the emergency situation to his experience as a missionary in Brazil, recalling that:

The measures that the Church in Italy has taken during this period are those that are always used when there are not enough priests in any other region of the world. We in Italy are used to customizing the funeral rite, but this is not possible all the time and in all places. In situations where this is not possible, the ritual is collective and very fast, and this does not seem traumatic.

Furthermore, according to Nicola, this exceptional suffering is caused by the fact that in the West, “we are used to well-being; we have removed the awareness of death and have hidden its concreteness. COVID-19 has suddenly put us in front of our own limits, and we realized that we are unable to face them.”

### Third Theme: Religion and Prayer as Sources of Resilience

Prayer was a central knot in the narratives of all the participants. According to Franco:

Prayer is the optimal instrument to get in touch with God and humanity. Prayer is not a trivial and impersonal formula, learned by the heart and which does not aim at the person. Prayer is an instrument to have a direct contact with God.

Many participants urged families to pray together in order to strengthen their sense of unity and the sharing of contact with the divine. As Giacomo pointed out: “Forced coexistence can find in prayer an opportunity for closeness, strengthened by looking into each other's eyes whilst addressing God. This empty space where even the experience of waiting feels equally void can become a significant experience.” Pope Francis compared the priests who assisted the COVID-19 patients to the shepherds who took care of their flock. In particular, one of the speeches most cited by the participants was the *Urbi et Orbi* blessing delivered by the Pope on March 27, 2020 in which the Holy Father referred to the figure of the boat in storm.^1^ As Domenico reported:

During the prayer of March 27th, the Pope compared the world population before COVID-19 to the situation described in the Gospel of Mark in which the disciples found themselves on board a boat during a storm. He used the metaphor of the storm to indicate the condition of vulnerability and the sense of bewilderment felt in the face of the explosion of the pandemic. He used the metaphor of “being all in the same boat” to emphasize the importance of building a sense of community and to say that it is not possible to save oneself alone. In this context, the Pope was able to instill hope and a sense of unity. The reference to the storm was very effective. It allowed us to give an image of our condition, of what we are all living together at the same time. What I then developed around that thought is the greater humility of the world around us.

Nicola, in this regard, shared that:

God does not remove the human condition of fragility. He only removed the situations of weakness in rare cases. Usually, he does not intervene to simplify the situation. He does not intervene, he leaves everything unchanged…except in rare cases. What really counts and must change is the way of looking at the situation on the part of those who live it, as for example Jesus did on the cross, reinterpreting the meaning of his death. God did not prevent him from experiencing death. Jesus had to reckon with death. He has remained, but instead of interpreting it as evil, he interpreted it as love.

## Discussion

During the first breakout of the COVID-19 pandemic, the category of Italian Catholic priests was among those most affected by the contagion (10). Similar to other studies on COVID-19 and the Ebola virus (38, 39), we therefore wanted to investigate how the priests lived this peculiar experience in that period and how they managed to handle the emergency vis-à-vis their relationships with their parishioners. The results showed that the first impact was shocking, but the fear for one's fate had immediately been replaced by the task of helping parishioners cope with stress. The narratives on the personal aspects were minimal. Rather, the priests gave the impression of not caring much to talk about their personal experiences nor the risks of the contagion they personally took. On the contrary, all the narrators immediately focused on the health emergency and regarded it as a precious and unmissable moment to reconsider their own existential and vocational path. This step was quickly and spontaneously associated with the concern to ensure that they provided support and assistance to their flock.

Many participants referred to the Pope as the source of inspiration for underlying the importance of the prayer. One of the most cited speeches was the one Pope Francis gave on March 27th, in which he mentioned the image of the boat in the storm. The participants appeared to have looked to the Pope as a shepherd of shepherds, a guide capable of transforming the emergence of a potentially overwhelming situation into an opportunity to strengthen community bonds and enable resilient strategies. This passage confirms what has already been indicated in the literature, namely, the ability of believers to deal with stressful situations in a particularly resilient way (22, 26, 40, 41). The possibility of including one's existential path within a divine design endowed with meaning—where events can be regarded as an expression of God's will—can be a source of resilience in the face of death anguish, as widely discussed by terror management theory (TMT) (42, 43). In the perspective of TMT, religion fulfills the need to alleviate the fear of death by giving coherence and significance to the dramatic events related to the evidence of death (44, 45). Considering the studies already present in the literature, religion allows the representation of death as a passage rather than annihilation, and this view responds to the need of individuals to give meaning to their lives (23, 41).

Scholars have already described how faith in religious contents helped people manage the stress caused by the COVID-19 pandemic (46, 47). In particular, the participants of our study appeared to be aware of the fact that they belonged to a category considered to be at risk. Thus, they reacted in a manner considered consistent with the TMT. The use of categories that deny death (such as the afterlife) allowed them to reduce anxiety and fear of death as well as deal with the stressful situations without paralyzing themselves. They have managed to handle negative emotions and transform them into motivation to provide social action and support for their flock. This capability to cope has guaranteed that they can safeguard a certain degree of their physical and mental well-being. In fact, even the restrictions caused by the epidemic have been interpreted as an opportunity to find time for inner reflection and prayer. Furthermore, the initiatives proposed and adopted by the parishes have made it possible to celebrate the liturgical acts with decidedly non-ordinary instruments and methods (e.g., streaming celebrations, telematic meetings of pastoral groups, etc.). This attitude helped the priests keep the religious sentiment alive, transforming their perception of powerlessness into active support for the whole community.

The funeral rite was highly underlined and confirmed by the participants. Its importance has been described as fundamental both for community life and for the mourning process, either familiar and individual, which is in accordance with the literature (18, 48). On the one hand, the priests' testimonies have highlighted the sufferings of the believers, specifically their inability to accompany their loved ones at the end of their lives (17). On the other hand, they underlined the urgent need to guarantee these mourners a special kind of support, which required an early and strong spiritual intervention. In spite of this, the overestimation by the faithful of the value of the funeral rite has also been criticized. It has been reported that, in emergency situations, even the simple collective blessing of the corpses is endowed with value, from the doctrinal point of view, as normally happens in mission areas in poor countries. We can recognize in this passage a further instrument of resilience: the reference to situations that are in a chronic state of emergency, as happens in the poorest areas of the planet, teaches the flock living in the richest countries (e.g., Italy) to consider their privileges and, therefore, bear the frustration of not being able to enjoy the psychological benefits that the traditional funeral ritual guarantees. This perspective highlights an important stance taken by priests: religion should not be considered a mere tool aimed at achieving individual psychological well-being. From this point of view, we must recognize that the power of resilience of religious discourse also hinges on the ability to renounce the subjective need for reassurance. Indeed, this kind of attitude can be useful but not necessarily comforting enough for all mourners, as highlighted in another study (18). In fact, in areas in Italy where the impact of the virus has been particularly violent, the need to ritualise the detachment and the loss has been managed via the Internet. As priests have managed to continue their pastoral activities, keeping alive the connection with their flock via the Internet, as revealed by the results of the current study and in the literature (49), it is our advice that they do the same with commemorative rituals. In doing so, they could support the mourners in the process of sharing their grief with special rites and sacraments, which could also help manage the trauma and close the mourning process. The ritual helps the mourners look ahead and return to normal life by elaborating the separation as a point of no return, instead of leaving the thought of loss constantly open, which is likely to happen if the mourner is left alone in reliving the grief via social media (50–52).

## Conclusions

The priests who participated in this research confirmed how religious faith has the power to transform fear and pain into motivation to strengthen the social bond, thus overcoming the individual need for reassurance. The transformation of the negative into positive can be seen as a reflection of the call to guide the community toward the goals of spirituality. The pastors enhanced family unity and closeness by indicating the ways of prayer and reflection about the finiteness of life. Therefore, from the point of view of the Catholic religion, the pandemic has provided opportunities to devote oneself to interiority and to share moments of reflection and prayer with loved ones.

Moreover, in order to help their parishioners, the priests quickly learnt to use digital tools to continue their pastoral activities (e.g., meetings with parishioners). In this regard, the Internet is likely to shape a new way of reaching the faithful in their homes, although—as pointed out by the participants—the availability of tools is currently limited. Nevertheless, the participants also highlighted the importance of the concrete, physical presence of the flock. Therefore, we suggest that it would be important to offer priests adequate training to help them enhance both their digital skills and the classical, ritual dimension linked to mourning and loss.

## Limitations and Future Prospects

As allowed by the guidelines of the IPA, the 12 participants were part of a selected and limited group of priests living in a specific area of Northern Italy that was strongly affected by the first phase of the pandemic. Therefore, inferences and generalizations were not permitted. Nevertheless, these results can be considered as a starting point for further investigations and explorations into the expansion of priestly communities in different areas of life as well as the roles and functions of the liturgy in different cultural and social contexts. Through the recruitment of a larger and more heterogeneous group of participants, an expanded version of this project could represent the initial phase of a longitudinal study investigating the evolution and modification of the psychological impact of the COVID-19 pandemic on this category of people. Finally, it would be very important to set up a quantitative study, using standardized questionnaires to survey the level of spirituality, work-related stress, moral distress and resilience. In fact, from the results that emerged from the present study, it appears that spirituality was the greatest source of resilience, and it would be important to survey the full spectrum of difficulties over which spirituality appears to be a strong protective factor.

## Data Availability Statement

The raw data supporting the conclusions of this article will be made available by the authors, without undue reservation.

## Ethics Statement

The studies involving human participants were reviewed and approved by The Ethical Committee for the Psychological Research of the University of Padua N.CD8470F7F42ACDD0679DC171884311B3. The patients/participants provided their written informed consent to participate in this study.

## Author Contributions

IT: project ideation, research design, supervision, analysis, article writing, and coordination. SZ: interviews, analysis, and article writing. EI: analysis, coordination, and supervision. CM: research design and supervision. PC: research design and supervision. KB: supervision. All authors contributed to the article and approved the submitted version.

## Conflict of Interest

The authors declare that the research was conducted in the absence of any commercial or financial relationships that could be construed as a potential conflict of interest.

## Footnotes

^1^Available online at: http://www.vatican.va/content/francesco/en/homilies/2020/documents/papa-francesco_20200327_omelia-epidemia.html
